# Physical Fitness and Cardiometabolic Disease

**DOI:** 10.31083/j.rcm2308259

**Published:** 2022-07-20

**Authors:** Peter Kokkinos, Jonathan Myers

**Affiliations:** ^1^Department of Kinesiology and Health, School of Arts and Sciences, Rutgers University, New Brunswick, NJ 08901, USA; ^2^Cardiology, Veterans Affairs Medical Center, Washington, DC 20422, USA; ^3^School of Medicine and Health Sciences, George Washington University, Washington, DC 20052, USA; ^4^Palo Alto Health Care System, Veterans Affairs Medical Center, Livermore, CA 94550, USA; ^5^Department of Cardiology, Stanford University, Stanford, CA 94305, USA

The increased energy demands of the muscular system at work require that several physiological systems adjust their functions accordingly to meet this demand. During this process, specific adaptations occur that make all systems involved more efficient in their respective role and more resilient to the wear and tear imposed by the increased demand. This results in the organism being more resilient to environmental stresses, injuries, diseases and demise. In short, the capacity to do physical work has played a crucial role in our survival.

This concept was described nearly 3000 years ago by the Greek physician Hippocrates, who stated that “…all parts of the body when used in the task they are accustomed to, become stronger and age slowly. If they remain idle, they become weaker, age quicker, succumb to disease and die.” Scientific scrutiny of the connection between physical activity (PA) and human health was first described by Professor Jeremy Morris and his coworkers [1] in the mid-20th century. In a series of studies, they demonstrated that the cardiovascular disease mortality rate of those performing physically demanding occupations, such as bus conductors and postal service workers, was roughly half of the rate experienced by bus drivers and desk clerks who had comparatively sedentary occupations. Approximately two decades later, a series of studies from the US by Paffenbarger and colleagues [2, 3] reported similar findings among workers with physically demanding occupations compared to their sedentary counterparts and college students participating in various sports. These and other early studies that followed, attempted to quantify PA by either the occupation of the participants or self-reported PA habits. Although both methods have inherent weaknesses, the findings of all these studies were strikingly similar to the original reports by Morris *et al*. [1], that PA is inversely related to all-cause and cardiovascular mortality. Furthermore, there was a dose-response relationship between the amount of PA and mortality risk. These concepts have been expanded in recent decades to include a spectrum of chronic conditions: regular PA has been consistently demonstrated to be inversely related to the incidence of cardiovascular, pulmonary, renal disease, cognitive decline, frailty, cancer, osteoporosis, diabetes and numerous other conditions [4].

During the eighties, a series of studies by Professor Steven Blair and colleagues emerged that shaped our understanding of the connection between PA and health [5, 6]. In these studies, fitness, and more specifically cardiorespiratory fitness (CRF), was assessed objectively by a standardized exercise treadmill test, therefore removing the ambiguity of self-reporting PA status. These seminal studies extended the understanding of the association between CRF and health have been replicated by many others [7] and have influenced guidelines on PA and health worldwide. For example, we now have a better understanding of the minimum exercise activity necessary for favorable outcomes [8] as well as the risks associated with excessive exercise practices [9]. Most recently, we have gained knowledge on the interaction between medications and PA in the prevention and management of chronic diseases [10, 11, 12]. The findings of these studies support the concept that increased PA is at least as effective as medications in the management of certain chronic diseases and often act synergistically with medications leading to enhance health outcomes.

Collectively, the overriding conclusion from the studies examining the association between CRF, PA and health outcomes is the existence of an inverse and graded association between the capacity to perform physical work, the incidence of disease and the risk of premature mortality [13, 14]. Moreover, this association is independent of comorbidities and is evident regardless of age, gender or race [13, 14, 15]. Despite the wealth of evidence regarding PA and health, PA remains underused as an intervention to reduce the incidence of disease and mortality risk [7]. This remains a challenge going forward. In this issue of RCM, content experts address chronic conditions and summarize the latest findings on the associations between PA, CRF and cardiometabolic disease.
